# Intrahepatic Tissue‐Resident CD8 ^+^ T‐Cell Programmes Associate With Fibrosis and Local Immune Remodelling in Autoimmune Hepatitis

**DOI:** 10.1111/liv.70897

**Published:** 2026-10-05

**Authors:** Victoria Stary, Sarah Losek, Magdalena Mairinger, Gerhard Prager, Jonas Santol, Patrick P. Starlinger, Thomas Reiberger, Albert F. Stättermayer, Georg Stary

**Affiliations:** ^1^ Division of Visceral Surgery, Department of General Surgery, Comprehensive Cancer Center Medical University of Vienna Vienna Austria; ^2^ Department of Surgery, HPB Center Vienna Health Network, Clinic Favoriten and Sigmund Freud Private University Vienna Austria; ^3^ Department of Surgery, Division of Hepatobiliary and Pancreas Surgery Mayo Clinic Rochester Minnesota USA; ^4^ Institute of Vascular Biology and Thrombosis Research, Center of Physiology and Pharmacology Medical University of Vienna Vienna Austria; ^5^ Division of Gastroenterology and Hepatology, Department of Medicine III Medical University of Vienna Vienna Austria; ^6^ Christian Doppler Laboratory for Portal Hypertension and Liver Fibrosis Medical University of Vienna Vienna Austria; ^7^ CeMM Research Center for Molecular Medicine of the Austrian Academy of Sciences Vienna Austria; ^8^ Department of Dermatology Medical University of Vienna Vienna Austria; ^9^ Christian Doppler Laboratory for Chronic Inflammatory Skin Diseases Vienna Austria

**Keywords:** autoimmune hepatitis, CD8 T cells, liver fibrosis, liver immunology, tissue residency, tissue‐resident memory T cells

## Abstract

**Background and Aims:**

Autoimmune hepatitis is an immune‐mediated liver disease characterized by chronic inflammation and progressive fibrosis. Tissue‐resident memory T cells mediate organ‐specific immunity, but their role in autoimmune hepatitis remains poorly understood. We characterized tissue‐residency‐associated T‐cell populations and their cytokine profiles, assessed associations with disease severity and determined whether these signatures were reflected in peripheral blood.

**Methods:**

Paired blood and liver samples were obtained from 19 patients with autoimmune hepatitis. Comparator cohorts included 20 patients with metabolic dysfunction‐associated steatotic liver disease and 20 surgical patients providing non‐inflamed adjacent non‐tumorous liver tissue. Multiparameter flow cytometry assessed tissue‐residency markers, T‐cell differentiation and cytokine production after polyclonal stimulation. Immune phenotypes were correlated with histological and clinical parameters.

**Results:**

Autoimmune hepatitis was associated with compartment‐specific remodelling of T‐cell differentiation states and intrahepatic enrichment of CD8^+^ T cells expressing CD69, CD103 and CXCR6 relative to paired blood and comparator liver tissues. Intrahepatic CD8^+^ T cells exhibited an interferon‐γ‐dominant cytokine profile, whereas CD4^+^ T cells showed broader interferon‐γ‐, interleukin‐17A‐ and interleukin‐4‐associated responses. Despite the enrichment of tissue‐residency‐associated CD8^+^ T cells, cytokine‐producing CD8^+^ T‐cell populations with and without a tissue‐resident memory phenotype contributed comparably to the hepatic T‐cell compartment. Tissue‐residency‐associated phenotypes correlated positively with Ishak fibrosis stage, whereas the frequency of interferon‐γ‐positive intrahepatic CD8^+^ T cells correlated inversely with fibrosis.

**Conclusions:**

Autoimmune hepatitis was characterized by intrahepatic accumulation of phenotypically distinct and functionally active CD8^+^ tissue‐resident memory T cells associated with fibrosis. This immune profile was not reflected in paired blood, indicating compartmentalized hepatic immune remodelling.

## Key Points

- Autoimmune hepatitis was characterized by intrahepatic enrichment of CD8+ T cells expressing tissue‐residency markers, a pattern not reflected in paired blood.
- Intrahepatic CD8+ T cells exhibited an interferon‐γ‐dominant cytokine profile, whereas CD4+ T cells showed broader cytokine responses.
- Intrahepatic CD3+ T cells with a tissue‐resident memory phenotype correlated positively with Ishak fibrosis stage, whereas interferon‐γ‐positive CD8+ T‐cell frequencies correlated inversely.
- Neither immune phenotype showed significant associations with serum ALT or histological inflammatory activity.

## Lay Summary

Autoimmune hepatitis is a rare disease in which the immune system attacks the liver, potentially causing progressive scarring, liver failure and the need for transplantation. Current treatments largely suppress the immune system broadly, making it important to better understand the immune cells involved. We found that a distinct group of local T cells in liver tissue was associated with inflammation and scarring, while these changes were not apparent in paired blood samples.

**Abbreviations**
AIH: autoimmune hepatitis
ALT: alanine aminotransferase
AMA: antimitochondrial antibodies
ANA: antinuclear antibodies
ANTL: adjacent non‐tumorous liver tissue
AST: aspartate aminotransferase
CCA: cholangiocarcinoma
CD: cluster of differentiation
CXCR6: C‐X‐C motif chemokine receptor 6
FBS: foetal bovine serum
FVD: fixable viability dye
HCC: hepatocellular carcinoma
HPB: hepatopancreatobiliary
iCCA: intrahepatic cholangiocarcinoma
IFN‐γ: interferon‐gamma
IgG: immunoglobulin G
IL: interleukin
IQR: interquartile range
LMNC: liver‐derived mononuclear cell
MASLD: metabolic dysfunction‐associated steatotic liver disease
mCRC: metastatic colorectal carcinoma
MFI: median fluorescence intensity
mHAI: modified Histology Activity Index
MMF: mycophenolate mofetil
NAS: NAFLD Activity Score
PBMC: peripheral blood mononuclear cell
PBS: phosphate‐buffered saline
PMA: phorbol 12‐myristate 13‐acetate
SAF: steatosis‐activity‐fibrosis
SD: standard deviation
SDC: simplified diagnostic criteria
SLA: soluble liver antigen antibody
SMA: smooth muscle antibodies
TCM‐like: central memory‐like T cell
TCR: T‐cell receptor
TEM‐like: effector memory‐like T cell
TEMRA‐like: terminally differentiated effector‐memory‐like T cell
TNF‐α: tumour necrosis factor‐alpha
TRM: tissue‐resident memory T cell

## 1 Introduction

Autoimmune hepatitis (AIH) is a chronic inflammatory liver disease affecting individuals of all ages and ethnicities, characterized by elevated serum transaminases and immunoglobulin G (IgG) levels, circulating autoantibodies and histologically by plasma cell‐rich interface hepatitis. Liver biopsy remains a central component of AIH diagnosis, assessment of inflammatory activity and fibrosis staging according to current international clinical practice guidelines [1, 2, 3]. Although these serological and histological features are key diagnostic hallmarks, accumulating evidence indicates that T cells are principal drivers of hepatic inflammation and tissue damage in AIH [4]. Histological analyses consistently reveal dense lymphocytic infiltrates dominated by CD4^+^ and CD8^+^ T cells, with cell numbers correlating with inflammatory activity and disease severity [5].

The liver constitutes a unique immunological environment that promotes immune cell retention and local immune surveillance [6]. In this context, tissue‐resident memory T cells have emerged as a distinct T‐cell population enriched within non‐lymphoid tissues and capable of rapid site‐specific immune responses [7, 8]. TRM cells are commonly defined by expression of residency markers such as CD69 and CD103 and exhibit transcriptional and functional programmes adapted to their tissue niche [9].

In the liver, TRM cells represent a substantial fraction of intrahepatic T cells and have been implicated in chronic inflammatory and autoimmune conditions [10]. However, their role in AIH is only beginning to be elucidated. A pivotal study by You et al. demonstrated a marked expansion of CD69^+^CD103^+^CD8^+^ TRM cells in liver tissue from AIH patients compared with other chronic liver diseases, including metabolic dysfunction‐associated steatotic liver disease (MASLD) and chronic hepatitis B [11]. These cells expressed elevated levels of cytotoxic and pro‐inflammatory mediators, including IFN‐γ and IL‐17A, and their frequency correlated with serum ALT, IgG levels, histological inflammation and fibrosis stage. Importantly, the abundance of hepatic CD8^+^ TRM cells declined following glucocorticoid therapy, suggesting a direct link to disease activity. Despite current immunosuppressive treatment strategies, including corticosteroids, azathioprine and mycophenolate mofetil, disease relapse and persistent inflammatory activity remain major clinical challenges in AIH [12, 13].

These findings position CD8^+^ TRM cells as potential key effector cells in AIH pathogenesis. CD4^+^ T cells are central orchestrators of autoimmune responses and differentiation into Th1, Th2 and Th17 lineages has been implicated in AIH pathophysiology [14, 15]. Recent multi‐omic work has further identified clonally expanded liver‐resident CD4+ T cells as a source of tumour necrosis factor that promotes auto‐aggressive CD8+ T‐cell responses in AIH [16]. Complementary multiparameter flow‐cytometric analyses of phenotypically defined tissue‐residency‐associated CD4+ and CD8+ T‐cell populations across paired liver and blood samples and comparator liver tissues may provide additional insight into their cytokine‐producing capacity and associations with histological disease features.

Unlike circulating T cells, TRM cells may serve as a persistent intrahepatic immune reservoir capable of driving relapse after treatment withdrawal [17]. Detailed characterization of hepatic CD4^+^ and CD8^+^ TRM cells in AIH may therefore improve our understanding of the relationship between local and peripheral immunity, provide insights into mechanisms sustaining chronic liver inflammation and facilitate the identification of immune signatures with potential relevance for disease diagnosis, monitoring and therapeutic stratification.

## 2 Methods

### 2.1 Patients and Tissue Material

Patients included in this study were recruited after written informed consent. AIH patients undergoing clinically indicated liver biopsy were enrolled in the study and recruited at the Medical University of Vienna. Diagnosis of autoimmune hepatitis was established by comprehensive clinicopathological assessment according to current international guidelines, including liver histology and calculation of the simplified International Autoimmune Hepatitis Group score [18, 19]. Control liver tissue (ANTL) was obtained from histologically non‐inflamed liver tissue distant from the pathological lesion in patients undergoing liver surgery for benign or malignant conditions at the Department of Surgery, HPB Center, Vienna Health Network, Clinic Favoriten. Patients with MASLD were recruited through clinically indicated liver biopsy or bariatric surgery at the Medical University of Vienna. Diagnosis was established by clinicopathological evaluation based on histological findings and the presence of metabolic risk factors. Patient characteristics for the AIH, MASLD and ANTL cohorts are summarized in Tables 1, 2, 3. Paired peripheral blood samples were collected at the time of tissue acquisition. Clinical and demographic parameters including age, sex, diagnosis and relevant clinical characteristics were recorded. All studies involving human material were conducted in accordance with the Declaration of Helsinki and were approved by the local ethics committee of the Medical University of Vienna (1262/2017 AIH, 2180/2020 MASLD, 2032/2013 ANTL).

**TABLE 1:** Clinical and demographic characteristics of the AIH study cohort.

ID	Sex	Age	Clinical setting at biopsy	SDC score	mHAI/Ishak fibrosis	Abs	Treatment at time of biopsy	IgG (mg/dL)	AST (U/L)	ALT (U/L)
AIH1	M	56	Active despite treatment	7/8	Grade: 2/18 Stage: 5/6	SMA	Prednisolone Azathioprine Cyclosporine A	1770	36	50
AIH2	M	61	Active despite treatment	6/8	Grade: 7/18 Stage: 6/6	ANA SMA	Prednisolone	1330	133	176
AIH3	F	68	Active despite treatment	5/8	Grade: 7/18 Stage: 6/6	ANA SMA	Prednisolone MMF	1300	151	144
AIH4	M	57	Treatment naïve	7/8	Grade: 8/18 Stage: 6/6	ANA SMA	None	1410	131	178
AIH5	M	34	Treatment naïve	5/8	Grade: 6/18 Stage: 2/6	None	None	1770	837	955
AIH6	F	39	Treatment naïve	7/8	Grade: 5/18 Stage: 1/6	ANA SLA	None	1790	44	60
AIH7	F	68	Active despite treatment	7/8	Grade: 11/18 Stage: 2/6	ANA SMA	Prednisolone MMF	2500	245	328
AIH8	F	74	Treatment naïve	7/8	Grade: 3/18 Stage: 6/6	ANA	None	1920	46	51
AIH9	F	73	Treatment naïve	7/8	Grade: 5/18 Stage: 3/6	ANA SMA	None	1820	249	307
AIH10	F	54	Treatment naïve	7/8	Grade: 7/18 Stage: 2/6	SMA AMA	None	2650	66	70
AIH11	M	28	Withdrawal assessment	6/8	Grade: 2/18 Stage: 2/6	None	Azathioprine	1310	21	22
AIH12	M	80	Treatment naïve	7/8	Grade: 8/18 Stage: 3/6	ANA SMA	None	1830	931	1785
AIH13	F	56	Treatment naïve	8/8	N/A	ANA SMA	None	2880	937	657
AIH14	M	48	Treatment naïve	7/8	Grade: 8/18 Stage: 2/6	ANA SMA	None	1620	342	699
AIH15	F	68	Active despite treatment	7/8	Grade: 7/18 Stage: 4/6	ANA SMA	Prednisolone Tacrolimus MMF	3040	231	305
AIH16	M	65	Withdrawal assessment	6/8	Grade: 2/18 Stage: 0/6	None	Azathioprine	690	21	14
AIH17	M	19	Treatment naïve	7/8	Grade: 2/18 Stage: 4/6	ANA SMA	None	2200	107	204
AIH18	F	71	Withdrawal assessment	7/8	Grade: 1/18 Stage: 1/6	None	Azathioprine	1020	22	15
AIH19	F	18	Active despite treatment	7/8	Grade: 4/18 Stage: 4/6	ANA	Prednisolone MMF	2650	81	11

Abbreviations: Abs, autoantibodies; AMA, antimitochondrial antibodies; ANA, antinuclear antibodies; mHAI, modified Histology Activity Index; MMF, mycophenolate mofetil; NA, mHAI inflammatory grade and Ishak fibrosis stage were not assessable due to extensive hepatic necrosis and loss of normal liver architecture; SDC, Simplified Diagnostic Criteria; SMA, smooth muscle antibodies.

**TABLE 2:** Clinicopathological characteristics of the MASLD cohort.

ID	Sex	Age	NAS	Histological activity (S/I/B)	Batts–Ludwig	Sample source
MASLD1	F	65	2/8	S: 1, I: 1, B: 0	4/4	Clinical biopsy
MASLD2	F	42	4/8	S: 2, I: 1, B: 1	3/4	Clinical biopsy
MASLD3	F	48	4/8	S: 3, I: 1, B: 0	2/4	Clinical biopsy
MASLD4	M	54	3/8	S: 1, I: 1, B: 1	4/4	Clinical biopsy
MASLD5	F	47	6/8	S: 2, I: 2, B: 2	3/4	Clinical biopsy
MASLD6	F	67	4/8	S: 1, I: 1, B: 2	3/4	Clinical biopsy
MASLD7	F	64	2/8	S: 1, I: 1, B: 0	4/4	Clinical biopsy
MASLD8	M	48	2/8	S: 1, I: 1, B: 0	1/4	Bariatric surgery
MASLD9	F	58	5/8	S: 2, I: 1, B: 2	1/4	Bariatric surgery
MASLD10	F	50	5/8	S: 3, I: 1, B: 1	0/4	Bariatric surgery
MASLD11	F	60	2/8	S: 1, I: 1, B: 0	0/4	Bariatric surgery
MASLD12	M	27	2/8	S: 1, I: 1, B: 0	2/4	Bariatric surgery
MASLD13	M	48	4/8	S: 2, I: 1, B: 1	0/4	Bariatric surgery
MASLD14	F	59	6/8	S: 3, I: 2, B: 1	1/4	Bariatric surgery
MASLD15	M	33	5/8	S: 3, I: 1, B: 1	3/4	Bariatric surgery
MASLD16	F	56	6/8	S: 3, I: 1, B: 2	3/4	Bariatric surgery
MASLD17	M	46	4/8	S: 2, I: 1, B: 1	NA	Bariatric surgery
MASLD18	M	49	1/8	S: 0, I: 1, B: 0	0/4	Bariatric surgery
MASLD19	M	47	3/8	S: 2, I: 1, B: 0	1/4	Bariatric surgery
MASLD20	F	43	3/8	S: 2, I: 1, B: 0	1/4	Bariatric surgery

Abbreviations: B, hepatocellular ballooning; Histological activity was assessed according to the SAF scoring system; I, lobular inflammation; NA, not assessable; NAS, NAFLD activity score; S, steatosis.

**TABLE 3:** Clinical characteristics of the ANTL control cohort.

ID	Sex	Age	Type of surgery	Diagnosis
ANTL1	M	77	Hemihepatectomy	HCC
ANTL2	M	77	Minor resection	Metastatic pancreatic carcinoma
ANTL3	F	44	Hemihepatectomy	mCRC
ANTL4	F	27	Minor resection	mCRC
ANTL5	M	64	Major resection	mCRC
ANTL6	F	67	Hemihepatectomy	CCA
ANTL7	F	62	Hemihepatectomy	iCCA
ANTL8	F	47	Major resection	mCRC
ANTL9	F	71	Hemihepatectomy	mCRC
ANTL10	M	69	Laparoscopic partial resection	HCC
ANTL11	M	71	Hemihepatectomy	mCRC
ANTL12	M	84	Laparoscopic partial resection	iCCA
ANTL13	M	87	Hemihepatectomy	mCRC
ANTL14	M	73	Minor resection	HCC
ANTL15	F	42	Hemihepatectomy	mCRC
ANTL16	M	58	Hemihepatectomy	mCRC
ANTL17	M	77	Laparoscopic partial resection	HCC
ANTL18	F	61	Hemihepatectomy	iCCA
ANTL19	F	59	Minor resection	mCRC
ANTL20	F	47	Minor resection	mCRC

Abbreviations: ANTL, adjacent non‐tumorous liver tissue; CCA, cholangiocarcinoma; HCC, hepatocellular carcinoma; iCCA, intrahepatic cholangiocarcinoma; mCRC, metastatic colorectal carcinoma.

### 2.2 Cell Isolation From Liver Tissue and Peripheral Blood

Fresh human liver tissue and paired peripheral blood samples were processed immediately after collection. Liver‐derived mononuclear cells were isolated by mechanical dissociation through 70‐μm cell strainers using phosphate‐buffered saline (PBS) supplemented with 10% foetal bovine serum (FBS). Peripheral blood mononuclear cells were isolated from paired blood samples by density‐gradient centrifugation using Ficoll–Paque according to standard protocols. Cells were washed and used directly for downstream flow‐cytometric analysis without cryopreservation unless otherwise indicated.

### 2.3 Flow Cytometry and Intracellular Cytokine Staining

For phenotypic and functional characterization, isolated liver‐derived and blood‐derived lymphocytes were stained with fluorochrome‐conjugated monoclonal antibodies listed in Table S1. For intracellular cytokine analysis, cells were stimulated ex vivo for 4 h using a cytokine activation cocktail containing phorbol 12‐myristate 13‐acetate (PMA) and ionomycin (Cytokine Activation Cocktail, BioLegend), in the presence of protein transport inhibitors, according to the manufacturer's instructions. Following stimulation, cells were stained for surface markers and subsequently fixed, permeabilized and stained for intracellular cytokines. Flow cytometric acquisition was performed on a Cytek Aurora spectral flow cytometer. Data were analysed using FlowJo software. A corresponding unstained control was included in each experiment. Viable leukocytes were defined as CD45^+^FVD eFluor 506^low^ events. The FVD cutoff was positioned at the density minimum separating FVD^low^ and FVD^high^ populations and was independently validated using a dead‐cell‐enriched freeze–thaw control and a 1:1 mixture of once‐ and twice‐thawed preparations (Figure S1A). T‐cell differentiation phenotypes were defined according to CD27 and CD45RA expression as CD27^+^CD45RA^+^ naïve‐like, CD27^+^CD45RA^−^ central‐memory‐like (TCM‐like), CD27^−^CD45RA^−^ effector‐memory‐like (TEM‐like) and CD27^−^CD45RA^+^ terminally differentiated effector‐memory‐like (TEMRA‐like) populations. Because CD95 was not included in the panel, CD27 + CD45RA+ T cells were classified as naïve‐like, as this population may also include stem cell memory T cells. TRM cells were defined as CD69^+^ and/or CD103^+^ cells. Intracellular production of IFN‐γ, IL‐4, IL‐13, IL‐17A and TNF‐α was quantified within the indicated T‐cell populations and subsets. TNF‐α was added to the intracellular cytokine panel after study initiation and was therefore assessed in a subset of samples.

### 2.4 Statistical Analysis

Statistical analyses were performed using GraphPad Prism 8 (GraphPad Software, San Diego, CA, USA). Unless otherwise indicated, data are presented as mean ± standard deviation (SD). Comparisons among three or more groups were conducted using the Kruskal–Wallis test followed by Dunn's multiple‐comparison test. Correlations, including associations of prespecified intrahepatic immune parameters with Ishak fibrosis stage, serum ALT and mHAI inflammatory grade, as well as associations of treatment status with serum ALT and AST, were assessed using Spearman's rank correlation coefficient. For exploratory analyses according to biochemical disease activity, patients were classified as biochemically active when ALT exceeded the laboratory upper limit of normal and as biochemically inactive when ALT was within the normal range. Differences between biochemically active and inactive patients were assessed using Mann–Whitney *U* tests. Histological scoring (mHAI inflammatory grade and Ishak fibrosis stage) was unavailable for one patient with autoimmune hepatitis because of extensive hepatic necrosis and loss of normal liver architecture. All statistical tests were two‐sided and statistical significance was defined as *p* < 0.05.

For Figures 3B and S5A, cytokine frequencies were summarized as cohort means for liver and peripheral blood. The ratio of the liver cohort mean to the peripheral‐blood cohort mean was calculated for each cytokine and cohort and log_2_‐transformed for heatmap visualization. The colour scale was centred at 0, with positive values indicating higher cytokine frequencies in liver and negative values indicating higher frequencies in peripheral blood. For Figure 4G, the proportional contributions of TRM and non‐TRM CD8^+^ T cells to cytokine production were calculated based on subset frequency and the frequency of cytokine‐positive cells within each subset. Samples without detectable cytokine‐positive events in both subsets were excluded from this analysis.

For Figure 4H, cytokine‐producing TRM and non‐TRM CD8^+^ T cells were expressed as percentages of total viable hepatic CD3^+^ T cells. These frequencies were derived from the proportion of CD8^+^ T cells among CD3^+^ T cells, the proportion of TRM or non‐TRM cells among CD8^+^ T cells, and the proportion of cytokine‐positive cells within the respective subset. Paired TRM and non‐TRM values were compared using Wilcoxon matched‐pairs signed‐rank tests with correction for multiple comparisons.

## 3 Results

### 3.1 AIH Is Associated With Compartment‐Specific Remodelling of Intrahepatic T‐Cell Differentiation States

To investigate immune alterations associated with AIH, we established a paired blood‐liver analysis workflow enabling direct comparison of circulating and intrahepatic T‐cell populations across three cohorts: patients with AIH (*n* = 19), patients with MASLD (*n* = 20) as a non‐autoimmune inflammatory liver control group and ANTL (*n* = 20) obtained distant from the pathological lesion in patients undergoing major liver surgery as non‐inflammatory liver controls (Figures 1A and S1A–C and Tables 1, 2, 3). The AIH cohort included 10 treatment‐naïve patients, six with active disease despite immunosuppression and three undergoing biopsy during treatment‐withdrawal assessment. Median ALT was 176 U/L (IQR 50.5–317.5), median IgG was 1790 mg/dL (IQR 1370–2350), median mHAI inflammatory grade was 5.5 (range 1–11) and median Ishak fibrosis stage was 3 (range 0–6). The MASLD cohort had a median NAFLD Activity Score of 4 (range 1–6) and median Batts–Ludwig fibrosis stage of 2 (range 0–4); ANTL samples were histologically non‐inflamed tissue. The gating strategy is shown in Figure 1B, and viable CD45^+^ leukocyte frequencies were comparable across cohorts in blood and liver (Figure S1B). The CD4/CD8 ratio differed between blood and liver across cohorts (Figure 1C). In liver, MASLD showed an increased CD4/CD8 ratio, increased CD4^+^ T‐cell frequencies and reduced CD8^+^ T‐cell frequencies compared with ANTL, whereas AIH was comparable to ANTL (Figure 1C–E). Blood CD4^+^ and CD8^+^ T‐cell frequencies were comparable across cohorts. Thus, overall CD4^+^ and CD8^+^ T‐cell composition was preserved in AIH, whereas MASLD showed a CD4‐dominated intrahepatic shift.

**FIGURE 1:**
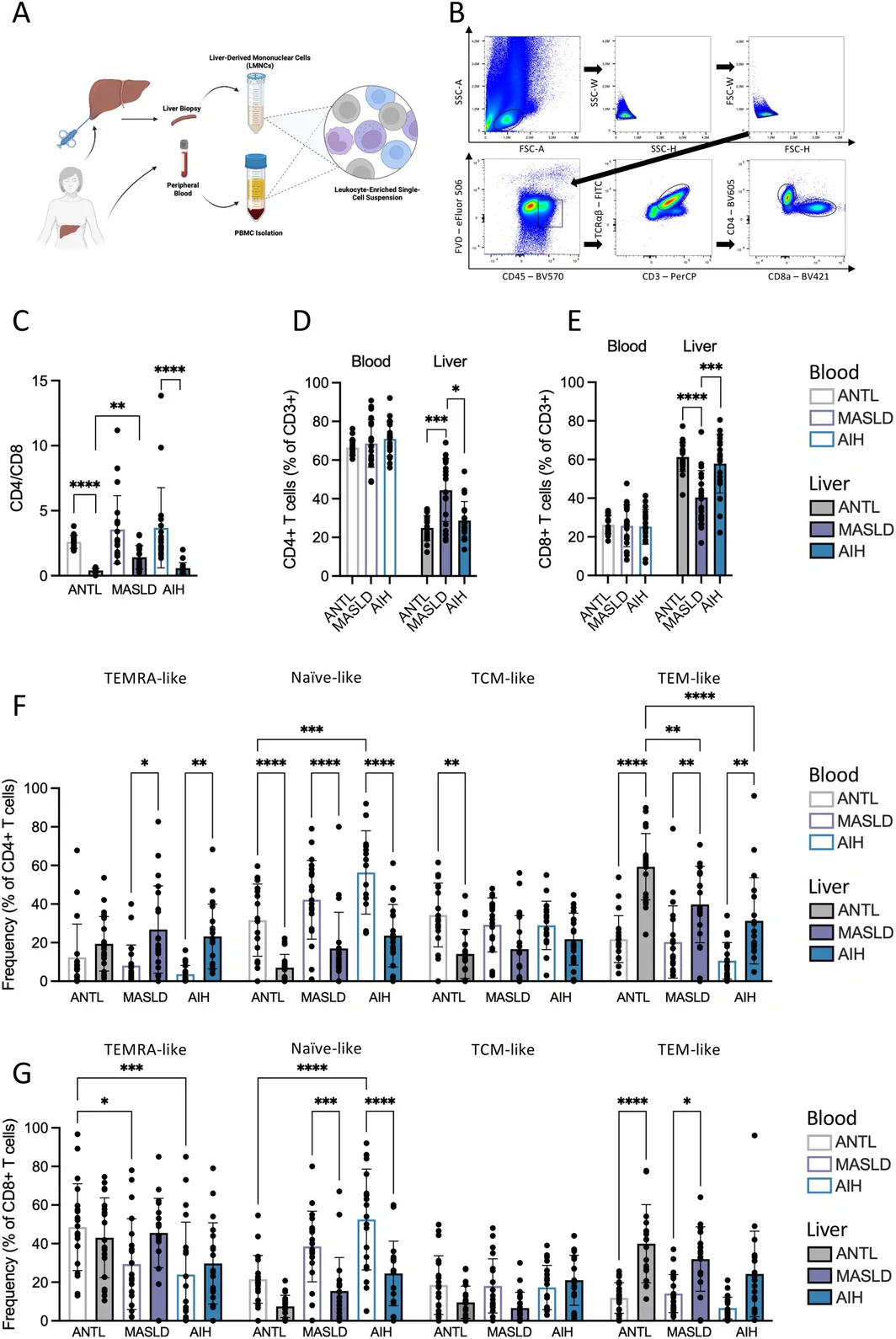
T‐cell composition and differentiation across blood and liver in ANTL, MASLD and AIH. (A) Study workflow showing paired analysis of peripheral blood mononuclear cells (PBMCs) and liver‐derived mononuclear cells (LMNCs). (B) Representative gating strategy for identification of viable CD45^+^CD3^+^ T cells and CD4^+^ and CD8^+^ T‐cell subsets. (C) CD4/CD8 ratio in peripheral blood and liver tissue. Frequencies of CD4^+^ (D) and CD8^+^ (E) T cells among CD3^+^ T cells in blood and liver. (F, G) CD27/CD45RA‐defined differentiation phenotypes of CD4^+^ (F) and CD8^+^ (G) T cells in peripheral blood and liver, operationally classified as CD27^+^CD45RA^+^ naïve‐like, CD27^+^CD45RA^−^ central‐memory‐like (TCM‐like), CD27^−^CD45RA^−^ effector‐memory‐like (TEM‐like) and CD27^−^CD45RA^+^ terminally differentiated effector‐memory‐like (TEMRA‐like) populations. Open bars represent peripheral blood and filled bars represent liver tissue. Bars represent mean ± SD with individual data points shown. Statistical comparisons are shown where indicated. Statistical significance was assessed using the Kruskal‐Wallis test followed by Dunn's multiple‐comparison test. *p < 0.05, **p < 0.01, ***p < 0.001, ****p < 0.0001.

As overall alterations in CD4^+^ and CD8^+^ T‐cell composition were modest in AIH, we next investigated whether disease‐associated changes were reflected in T‐cell differentiation phenotypes. T cells were classified according to CD27 and CD45RA expression as CD27^+^CD45RA^+^ naïve‐like, CD27^+^CD45RA^−^ TCM‐like, CD27^−^CD45RA^−^ TEM‐like and CD27^−^CD45RA^+^ TEMRA‐like populations (Representative gating: Figure S1C). Because CD95, CCR7 and CD62L were not included in the panel, these populations are described as phenotypically defined ‘‐like’ subsets rather than canonical naïve and memory T‐cell populations. Across all study groups, marked differences were observed between blood and liver, with liver‐derived CD4^+^ and CD8^+^ T cells exhibiting a more differentiated phenotype characterized by reduced frequencies of naïve‐like cells and increased proportions of TEMRA‐like and TEM‐like subsets (Figure 1F,G). Against this background, AIH was associated with a relative shift towards less differentiated T‐cell phenotypes. Within the CD4^+^ compartment, AIH liver samples displayed increased frequencies of naïve‐like T cells and reduced TEM‐like populations compared with ANTL, whereas TCM‐like and TEMRA‐like frequencies remained largely unchanged (Figure 1F). Similarly, AIH was associated with increased frequencies of naïve‐like CD8^+^ T cells in the liver (Figure 1G). Comparable changes were also observed in peripheral blood, where AIH patients exhibited increased frequencies of naïve‐like CD4^+^ and CD8^+^ T cells together with reduced CD8^+^ TEMRA‐like frequencies. Collectively, these findings indicate that AIH is associated with a shift towards less differentiated CD27/CD45RA‐defined T‐cell phenotypes that is evident both within the liver and in the circulation.

### 3.2 Tissue‐Resident and CXCR6 ^+^ T‐Cell Populations Accumulate Within the AIH Liver Microenvironment

Because AIH‐associated changes were primarily hepatic, we assessed CD69 and CD103 expression on circulating and liver‐derived CD4^+^ and CD8^+^ T cells (Figures 2A–C and S2A,B). Compared with blood, liver‐derived CD8^+^ T cells were enriched for CD69 single‐positive, CD103 single‐positive and CD69^+^CD103^+^ double‐positive populations. AIH liver tissue contained higher frequencies of CD69^+^CD103^+^ and CD103^+^CD69^−^ CD8^+^ T cells and fewer double‐negative cells than ANTL (Figure 2B), whereas liver‐derived CD4^+^ T cells showed no comparable coordinated pattern (Figure 2A). Circulating CD4^+^ and CD8^+^ T cells were predominantly CD103^−^CD69^−^ without significant differences between cohorts (Figure S2A,B). CXCR6^+^ CD4^+^ and CD8^+^ T cells were strongly enriched in liver compared with paired blood across cohorts, with the highest frequencies in MASLD and AIH (Figure 2D,E). Thus, AIH was characterized by compartment‐specific accumulation of tissue‐residency‐associated CD8^+^ T‐cell populations, while increased hepatic CXCR6 expression indicated local tissue adaptation in both CD4^+^ and CD8^+^ compartments.

**FIGURE 2:**
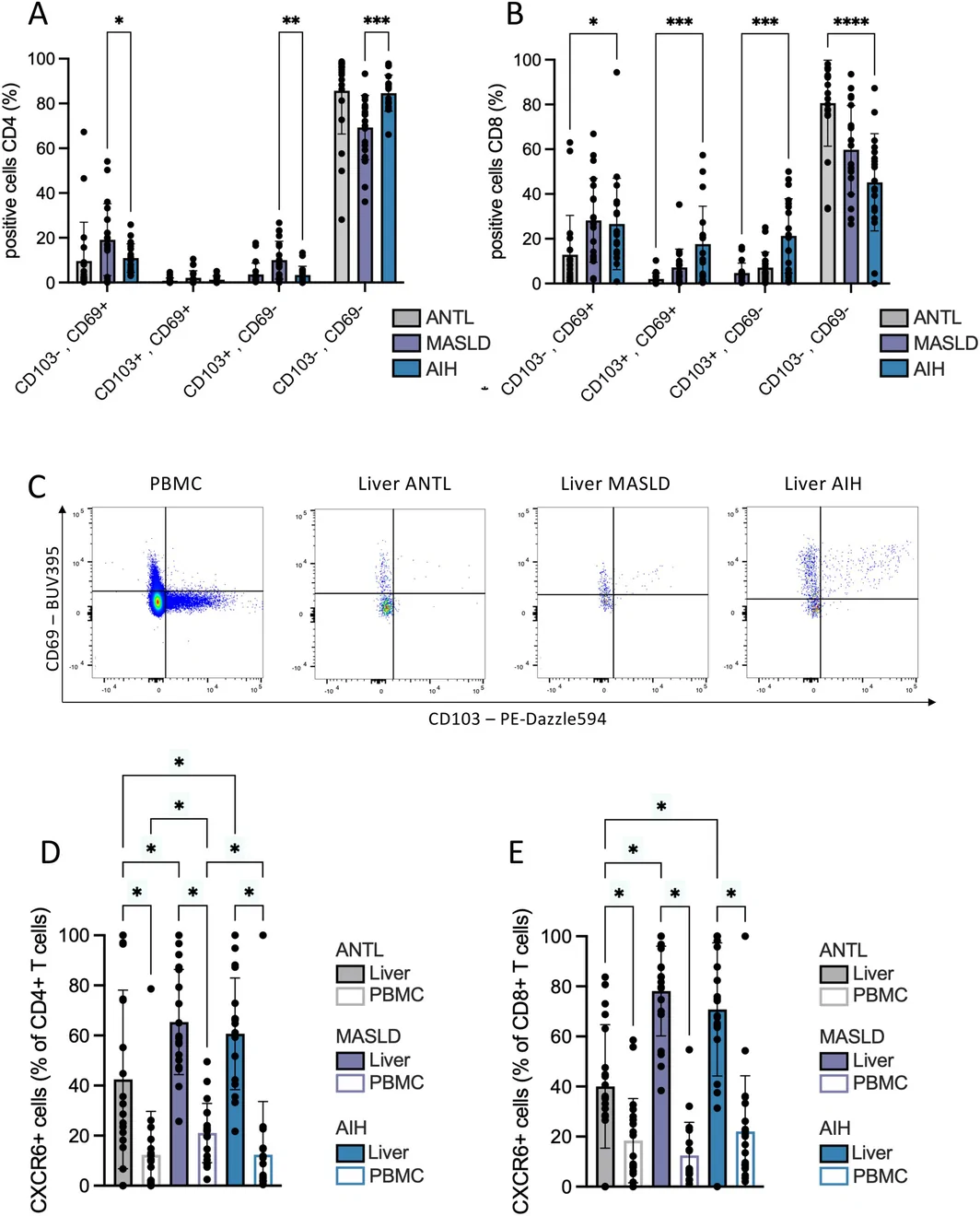
Tissue‐residency‐associated phenotypes of intrahepatic T cells in ANTL, MASLD and AIH. Distribution of CD69/CD103‐defined subsets among liver‐derived CD4^+^ (A) and CD8^+^ (B) T cells from ANTL, MASLD and AIH patients. Subsets were classified as CD69 single‐positive (CD103^−^CD69^+^), CD69^+^CD103^+^ double‐positive, CD103 single‐positive (CD103^+^CD69^−^) and double‐negative (CD103^−^CD69^−^) cells. (C) Representative flow‐cytometry plots showing CD69 and CD103 expression on circulating and intrahepatic CD8^+^ T cells. Frequencies of CXCR6^+^ cells among CD4^+^ (D) and CD8^+^ (E) T cells in blood and liver tissue. Data represent mean ± SD, with individual patient values shown. Statistical significance was assessed using the Kruskal–Wallis test followed by Dunn's multiple‐comparison test. *p < 0.05, **p < 0.01, ***p < 0.001, ****p < 0.0001.

### 3.3 Distinct Cytokine Signatures Characterize Intrahepatic CD4 ^+^ and CD8 ^+^ T‐Cell Responses in AIH

To further characterize the functional polarization of circulating and liver‐derived T‐cell populations, we analysed cytokine production following short‐term polyclonal stimulation. Representative histograms illustrated intracellular IFN‐γ staining in liver‐derived CD8^+^ T cells across the ANTL, MASLD and AIH cohorts (Figure 3A), complemented by representative histograms for IL‐17A, TNF‐α, IL‐4 and IL‐13 in Figure S3. The liver‐to‐blood distribution of cytokine‐producing cells was summarized across cytokines and cohorts in heatmaps, revealing broadly overlapping patterns in the CD8^+^ and CD4^+^ T‐cell compartments, with subset‐ and cohort‐dependent differences in magnitude (Figures 3B and S5A). Detailed quantitative analysis of CD8^+^ T cells identified prominent IFN‐γ production by liver‐derived populations from patients with AIH (Figure 3C). TNF‐α production was detectable in both liver‐derived and circulating CD8^+^ T cells, with descriptively higher frequencies in AIH than in ANTL but no significant AIH‐associated differences (Figure 3G). MASLD samples exhibited comparatively stronger IL‐17A‐associated responses (Figure 3D), whereas IL‐4 and IL‐13 responses were detectable but generally less prominent within the CD8^+^ T‐cell compartment (Figure 3E,F). In liver‐derived CD8^+^ T cells, IFN‐γ median fluorescence intensity was higher in MASLD than in ANTL, whereas the other cytokines did not differ significantly between cohorts (Figure S4). Compared with CD8^+^ T cells, CD4^+^ T cells displayed a more diverse pattern of cytokine responses. Liver‐derived CD4^+^ T cells from patients with AIH exhibited increased IFN‐γ and IL‐17A responses (Figure S5B,C). Intrahepatic CD4^+^ T cells also displayed substantial IL‐4 production, whereas IL‐13 responses remained comparatively low across cohorts and compartments (Figure S5D,E). TNF‐α was detectable within the CD4^+^ T‐cell compartment and appeared more liver‐skewed in AIH, although the corresponding AIH‐associated differences were not statistically significant (Figure S5F). Together, these findings indicate an IFN‐γ‐associated inflammatory signature shared across the intrahepatic CD8^+^ and CD4^+^ T‐cell compartments, accompanied by broader IL‐17A‐ and IL‐4‐associated responses among CD4^+^ T cells.

**FIGURE 3:**
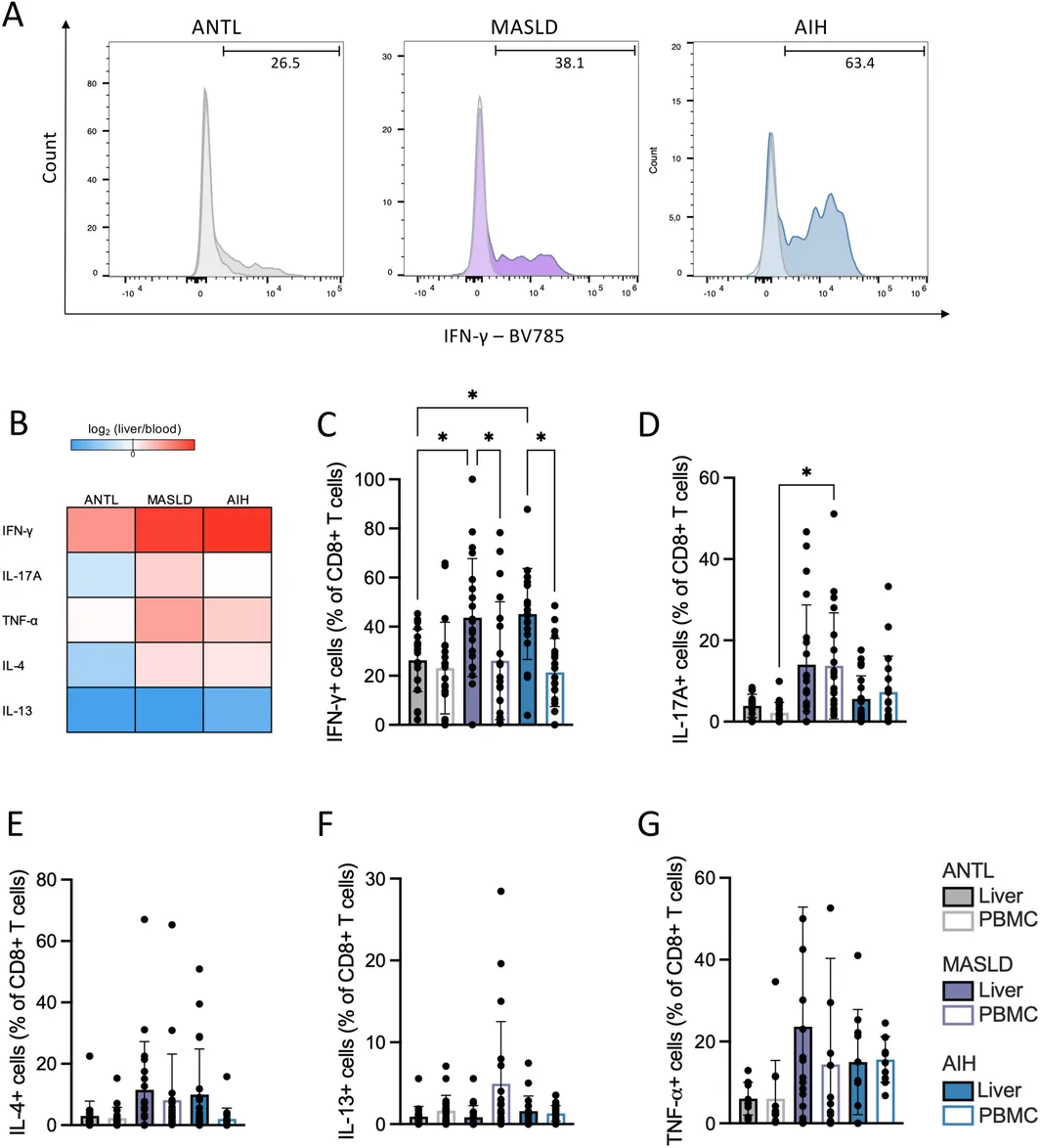
Pro‐inflammatory cytokine responses in circulating and intrahepatic CD8^+^ T cells. (A) Representative flow‐cytometry histograms showing intracellular IFN‐γ staining in liver‐derived CD8^+^ T cells following PMA/ionomycin stimulation. (B) Heatmap showing log_2_‐transformed ratios of cohort‐mean cytokine‐positive CD8+ T‐cell frequencies in liver versus peripheral blood across ANTL, MASLD and AIH cohorts; positive values indicate higher mean frequencies in liver and negative values indicate higher mean frequencies in peripheral blood. (C‐G) Frequencies of IFN‐γ^+^ (C), IL‐17A^+^ (D), IL‐4^+^ (E), IL‐13^+^ (F) and TNF‐α^+^ (G) CD8^+^ T cells in blood and liver tissue. Cytokine‐positive cells are expressed as percentage of CD8^+^ T cells. Data represent mean ± SD with individual patient values shown. Statistical significance was assessed using the Kruskal–Wallis test followed by Dunn's multiple‐comparison test. *p < 0.05, **p < 0.01, ***p < 0.001, ****p < 0.0001.

### 3.4 AIH‐Associated CD8 ^+^ TRM Cells Contribute to Intrahepatic Inflammatory Cytokine Programmes

To further investigate the functional contribution of tissue‐resident T cells in AIH, we compared cytokine production between TRM and non‐TRM CD8^+^ T‐cell populations isolated from liver tissue across ANTL, MASLD and AIH cohorts (Figure 4A). Given the marked enrichment of tissue‐residency‐associated CD8^+^ T‐cell populations observed in Figure 2, we sought to determine whether these cells contribute preferentially to specific intrahepatic cytokine programmes. Comparison of cytokine frequencies within TRM and non‐TRM populations revealed only modest differences across disease groups. IFN‐γ production was detectable in both compartments, whereas IL‐17A, IL‐13, IL‐4 and TNF‐α responses showed variable distributions across cohorts (Figure 4B–F). Collectively, these findings suggested that AIH‐associated TRM cells are not characterized by uniformly enhanced cytokine production compared with non‐TRM populations. To resolve the subset‐specific contribution to intrahepatic cytokine responses in AIH, we quantified the relative representation of TRM and non‐TRM cells within the total cytokine‐positive CD8^+^ T‐cell pool for each cytokine (Figure 4G). This analysis revealed cytokine‐dependent variation in the relative representation of TRM and non‐TRM cells, as exemplified by the greater relative contribution of TRM cells to the IFN‐γ‐positive CD8^+^ T‐cell pool. An additional analysis quantified the percentages of all viable hepatic CD3^+^ T cells that were cytokine‐positive CD8^+^ TRM cells or cytokine‐positive CD8^+^ non‐TRM cells (Figure 4H). Although mean frequencies of IFN‐γ‐positive and TNF‐α‐positive non‐TRM cells were numerically higher than those of the corresponding TRM populations, none of the paired comparisons reached statistical significance. Collectively, these analyses indicate that both TRM and non‐TRM CD8^+^ T cells contribute to intrahepatic cytokine responses in AIH, without demonstrating a consistently predominant contribution by either subset.

**FIGURE 4:**
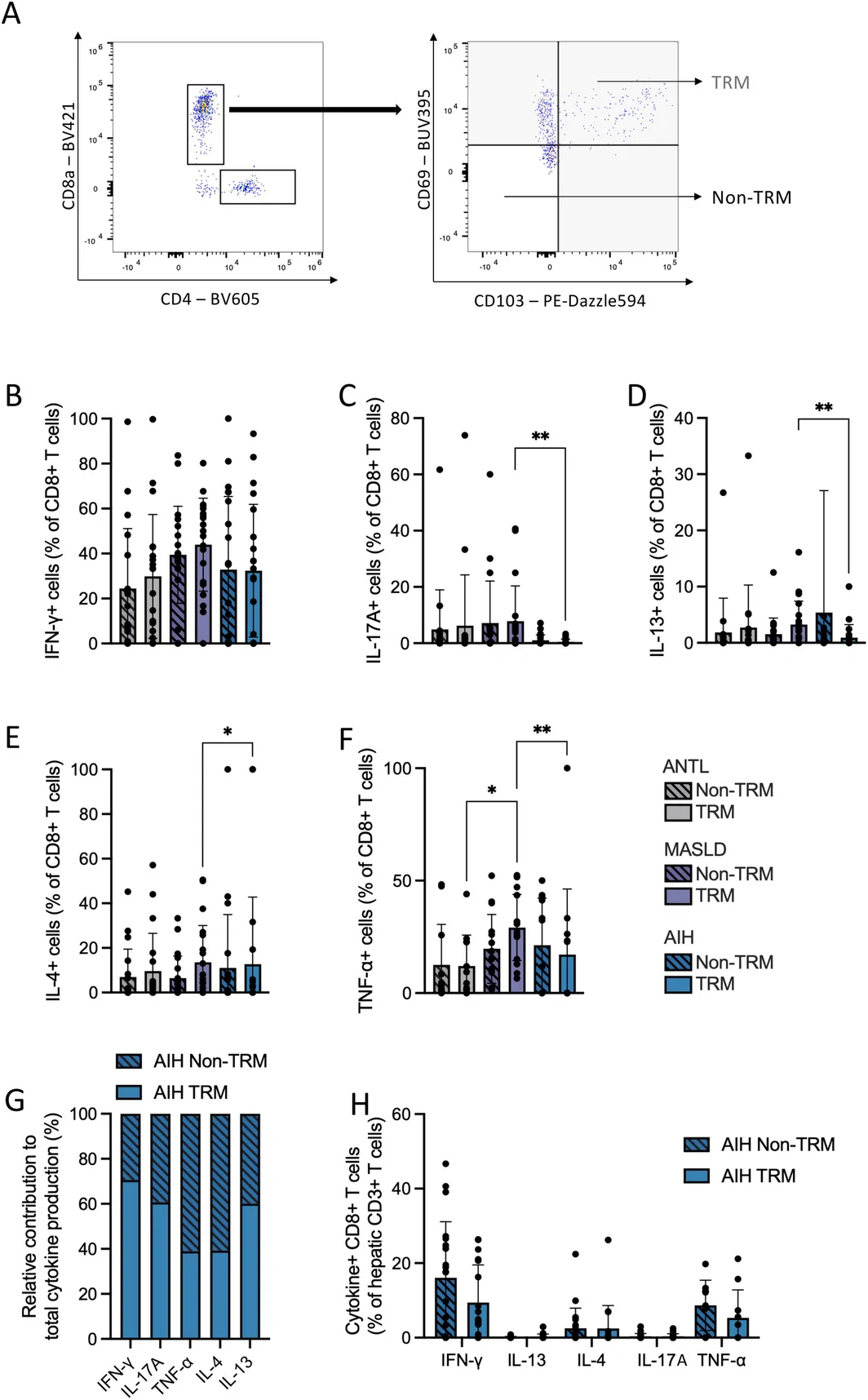
Functional cytokine profile of tissue‐residency‐associated and non‐resident CD8^+^ T cells in liver tissue. (A) Representative gating strategy for identification of tissue‐residency‐associated (CD69^+^ and/or CD103^+^) and non‐resident (CD69^−^CD103^−^) CD8^+^ T cells. Frequencies of IFN‐γ^+^ (B), IL‐17A^+^ (C), IL‐13^+^ (D), IL‐4^+^ (E) and TNF‐α^+^ (F) cells within tissue‐residency‐associated and non‐resident CD8^+^ T‐cell populations in liver tissue from ANTL, MASLD and AIH patients. (G) Relative contribution of tissue‐residency‐associated and non‐resident CD8^+^ T cells to cytokine‐producing CD8^+^ T‐cell populations in AIH liver tissue. (H) Frequencies of cytokine‐producing tissue‐residency‐associated and non‐resident CD8+ T cells expressed as percentages of total viable hepatic CD3+ T cells in AIH liver samples. Data are shown as mean ± SD with individual patient values. Statistical significance in B‐F was assessed using Kruskal–Wallis tests followed by Dunn's multiple‐comparison test. Paired TRM and non‐TRM values in H were compared using Wilcoxon matched‐pairs signed‐rank tests with correction for multiple comparisons. *p < 0.05, **p < 0.01, ***p < 0.001, ****p < 0.0001.

### 3.5 Tissue‐Residency‐Associated Immune Signatures Correlate With Histological Fibrosis Severity in Autoimmune Hepatitis

To explore the potential clinical relevance of the tissue‐resident immune signatures identified in previous analyses, we next examined the relationship between intrahepatic TRM‐associated T‐cell phenotypes and biochemical as well as histopathological markers of disease activity in AIH patients. Clinical characteristics of the AIH cohort are summarized in Table 1. As expected, immunosuppressive treatment was associated with lower transaminase levels, with a significant association observed for ALT (Figure S6A) and a similar, although non‐significant, trend for AST (Figure S6B).

Given the pronounced enrichment of intrahepatic TRM‐associated phenotypes observed above, we next investigated whether these immune alterations correlate with histological disease severity. Interestingly, the frequency of intrahepatic TRM‐phenotype CD3^+^ T cells positively correlated with Ishak fibrosis stage in AIH patients (Spearman's *ρ* = 0.589, *p* = 0.0101; Figure 5A), indicating that accumulation of tissue‐resident T‐cell populations is associated with more advanced chronic liver injury and fibrotic remodelling. In contrast, frequencies of IFN‐γ‐producing intrahepatic CD8^+^ T cells demonstrated an inverse correlation with Ishak fibrosis stage (Spearman's *ρ* = −0.744, *p* = 0.0004; Figure 5B), suggesting that progressive fibrosis may be accompanied by altered inflammatory T‐cell functionality within the hepatic microenvironment.

**FIGURE 5:**
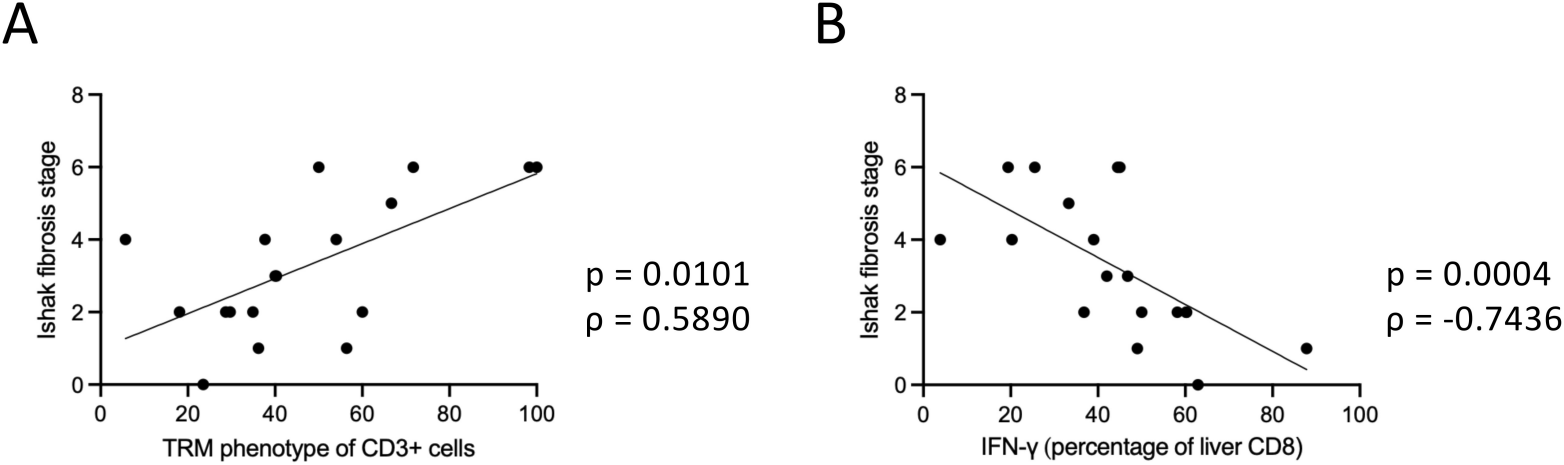
Associations of intrahepatic T‐cell phenotypes with Ishak fibrosis stage in AIH. (A) Correlation between intrahepatic tissue‐resident memory T‐cell (TRM) frequency and Ishak fibrosis stage. (B) Correlation between IFN‐γ‐producing intrahepatic CD8^+^ T cells and Ishak fibrosis stage. Each dot represents one patient. Spearman's rank correlation coefficients (*ρ*) and corresponding *p* values are shown. Statistical significance was assessed using Spearman's rank correlation coefficient.

To determine whether these fibrosis‐associated immune signatures were also related to contemporaneous inflammatory disease activity, we additionally examined their associations with serum ALT and histological activity as assessed by the modified Histology Activity Index (mHAI). The frequency of intrahepatic TRM‐phenotype CD3^+^ T cells was not significantly associated with ALT (*ρ* = −0.277, *p* = 0.251) or mHAI inflammatory grade (*ρ* = 0.065, *p* = 0.798). Similarly, the frequency of IFN‐γ‐producing intrahepatic CD8+ T cells showed no significant correlation with ALT (*ρ* = 0.072, *p* = 0.770) or mHAI inflammatory grade (*ρ* = 0.116, *p* = 0.647) (Figure S7A–D).

We further performed an exploratory analysis according to biochemical activity at the time of biopsy. Patients with ALT above the laboratory upper limit of normal were classified as biochemically active (*n* = 15), whereas patients with ALT within the normal range were classified as biochemically inactive (*n* = 4). Neither the frequency of intrahepatic TRM‐phenotype CD3^+^ T cells nor the frequency of IFN‐γ‐producing intrahepatic CD8^+^ T cells differed significantly between biochemically active and inactive patients (*p* = 0.262 and *p* = 0.736, respectively; Figure S7E,F). Given the small biochemically inactive subgroup and heterogeneous treatment status, these subgroup comparisons should be considered exploratory.

Together, these findings identify associations between intrahepatic tissue‐residency‐associated immune phenotypes and histological fibrosis severity in AIH, whereas corresponding associations with contemporaneous biochemical or histological inflammatory activity were not detected in this cohort.

## 4 Discussion

In this study, paired phenotypic and functional profiling of intrahepatic and circulating T cells across AIH, MASLD and ANTL identified three principal findings. First, AIH was characterized by enrichment of intrahepatic CD8^+^ T cells with a CD69‐ and/or CD103‐defined tissue‐residency‐associated phenotype that was largely absent from paired blood. Second, intrahepatic CD8^+^ T cells in AIH showed predominantly IFN‐γ‐associated cytokine responses, whereas CD4^+^ T cells displayed a broader repertoire including IFN‐γ, IL‐17A and IL‐4. Third, TRM‐associated immune signatures correlated with fibrosis stage. Together, these findings identify compartmentalized, tissue‐adapted T‐cell programmes associated with chronic hepatic immune remodelling in AIH [8, 11].

Our findings extend and complement the pivotal study by You et al., which first identified hepatic CD69^+^CD103^+^CD8^+^ TRM expansion in AIH and demonstrated associations with ALT, IgG levels, inflammatory activity and fibrosis stage [11]. Importantly, while You et al. primarily focused on phenotypic characterization and cytotoxic functionality, our study additionally integrates paired blood‐liver analyses and functional cytokine profiling across both CD4^+^ and CD8^+^ compartments. The marked enrichment of TRM‐like cells within liver tissue together with their scarcity in peripheral blood strongly supports the concept that disease‐relevant immune programmes in AIH are maintained locally within hepatic tissue rather than reflected by circulating immune compartments. Increased CXCR6 expression in intrahepatic T cells further supports tissue‐retention programmes associated with local immune surveillance. In AIH, these tissue‐adapted programmes may become pathologically dysregulated and contribute to sustained autoimmune tissue injury [20].

Functionally, our data demonstrate that intrahepatic CD8^+^ T cells in AIH exhibit dominant IFN‐γ‐associated inflammatory polarization. This finding is biologically plausible given the established role of IFN‐γ‐driven cytotoxic immune responses in hepatocyte injury and autoimmune inflammation [14, 15]. Importantly, the CD4+ compartment showed a broader cytokine profile, with IFN‐γ‐, IL‐17A‐ and IL‐4‐associated responses, indicating that intrahepatic CD4+ T cells are functionally involved in the local inflammatory milieu rather than representing a bystander population. These observations are particularly relevant in light of the recent study by Xu et al. [16], who used integrative multi‐omics and functional analyses to identify a local immune network in AIH in which myeloid‐ and hepatocyte‐derived IL‐15 promotes proliferation and cytotoxicity of liver auto‐aggressive CD8^+^ T cells, while TNF derived from clonally expanded liver‐resident CD4^+^ T cells licences their full cytotoxic programme. Our paired protein‐level data complement this mechanistic framework by demonstrating pronounced hepatic compartmentalization of inflammatory CD8^+^ and CD4^+^ T‐cell programmes. Because TNF‐α was introduced into the panel after study initiation, it was assessed in a smaller subset of samples, limiting statistical power for cohort‐ and compartment‐specific comparisons. The accompanying phase IIa infliximab trial further highlights the translational relevance of this axis, although these early clinical findings require validation in larger controlled studies. Within the CD8^+^ compartment, cytokine production was distributed across both TRM and non‐TRM populations. When cytokine‐producing cells were expressed relative to the total hepatic CD3^+^ T‐cell compartment, neither population significantly dominated the analysed IFN‐γ, IL‐13, IL‐4, IL‐17A or TNF‐α responses. These findings indicate functional participation of tissue‐residency‐associated CD8+ T cells in the intrahepatic inflammatory response without suggesting that they constitute an exclusively dominant source of cytokine production. An important aspect of our study is the observation that TRM‐associated immune signatures correlated positively with fibrosis stage. While causality cannot be established from our data, this association suggests that persistent tissue‐resident immune activation may contribute to progressive tissue remodelling and chronic liver damage in AIH. This finding is consistent with You et al., who also reported associations between hepatic CD69^+^CD103^+^CD8^+^ TRM cells and histological inflammation and fibrosis [11]. In contrast to the associations with inflammatory activity reported by You et al., neither the TRM‐associated phenotype nor IFN‐γ‐producing intrahepatic CD8^+^ T cells showed significant associations with serum ALT or mHAI inflammatory grade in our cohort. This pattern raises the possibility that the fibrosis‐associated immune signatures identified here may reflect aspects of chronic tissue remodelling rather than contemporaneous inflammatory activity alone. However, given the limited cohort size, heterogeneous treatment status and small number of biochemically inactive patients, these findings should not be interpreted as demonstrating independence from ongoing disease activity. Interestingly, IFN‐γ‐producing CD8^+^ T cells inversely correlated with fibrosis stage, potentially reflecting dynamic changes in immune composition and functional exhaustion during advanced disease progression.

Alternatively, progressive fibrotic remodelling itself may alter the hepatic immune microenvironment and reduce inflammatory cytokine production. Longitudinal studies will be required to determine whether TRM‐associated immune states directly promote fibrogenesis or instead represent markers of chronic inflammatory persistence.

Clinically, our findings suggest that the disease‐relevant immune compartment in AIH may not be adequately captured by routine blood‐based immune monitoring. In our cohort, TRM‐associated phenotypes were markedly enriched within the liver but were largely absent from peripheral blood, supporting the concept that key pathogenic immune programmes in AIH are maintained locally within hepatic tissue rather than reflected systemically. Although our current data did not identify a robust circulating surrogate for intrahepatic TRM activity, higher‐resolution paired blood‐liver approaches may reveal rare tissue‐imprinted circulating populations. Longitudinal studies integrating single‐cell transcriptomics, T‐cell receptor repertoire analysis and treatment‐response profiling will be required to determine whether such signatures can ultimately serve as non‐invasive surrogates of pathogenic intrahepatic immune activity.

Several limitations should be acknowledged. The cross‐sectional design precludes causal inference and the cohort size, particularly the four biochemically inactive patients, limited subgroup comparisons. ANTL controls were histologically non‐inflamed samples collected distant from surgical lesions, but effects of the underlying disease or prior treatment cannot be excluded. The AIH cohort included different disease stages and treatment states, contributing to biological heterogeneity. Although age distributions were broadly comparable, age‐related effects on T‐cell differentiation may have influenced intergroup differences. Cytokine responses after short‐term PMA/ionomycin stimulation reflect inducible potential rather than direct ex vivo activity. Tissue residency was defined phenotypically by CD69/CD103 without functional confirmation; autoreactivity and antigen specificity were not assessed. Future clonotype‐resolved, spatial and longitudinal studies will be needed to define TRM heterogeneity and treatment‐related remodelling [21, 22].

Collectively, our study identifies intrahepatic CD8^+^ T cells with a tissue‐residency‐associated phenotype as a functionally active compartment associated with chronic inflammatory remodelling in AIH. Locally retained CD8^+^ and CD4^+^ T‐cell programmes may contribute to persistent hepatic immune activation that is incompletely reflected in the circulation, providing a basis for future tissue‐focused mechanistic and biomarker studies.

## Author Contributions

**Victoria Stary:** conceptualization, methodology, investigation, formal analysis, data curation, visualization, writing – original draft, writing – review and editing. **Sarah Losek:** investigation, methodology, data curation, writing – review and editing. **Magdalena Mairinger:** resources, writing – review and editing. **Gerhard Prager:** resources, writing – review and editing. **Jonas Santol:** resources, writing – review and editing. **Patrick P. Starlinger:** resources, writing – review and editing. **Thomas Reiberger:** resources, writing – review and editing. **Albert F. Stättermayer:** conceptualization, resources, validation, writing – review and editing. **Georg Stary:** supervision, conceptualization, validation, writing – review and editing. All authors approved the final version of the manuscript.

## Funding

The authors have nothing to report.

## Ethics Statement

Patients included in this study were recruited after written informed consent. All studies involving human material were conducted in accordance with the Declaration of Helsinki and were approved by the local ethics committee of the Medical University of Vienna (1262/2017 AIH, 2180/2020 MASLD, 2032/2013 ANTL).

## Conflicts of Interest

The authors declare no conflicts of interest.

## Supporting information


**Figure S1:** Viability gating, leukocyte recovery and CD27/CD45RA‐defined T‐cell differentiation phenotypes. (A) Experimental validation of the viability‐dye gate. Representative CD45/FVD eFluor 506 plots from an independent validation experiment after one freeze–thaw cycle, after an additional freeze–thaw cycle and following a 1:1 mixture of both preparations. (B) Frequencies of viable CD45^+^ leukocytes in peripheral blood and liver tissue from ANTL, MASLD and AIH patients. Bars represent mean ± SD, with individual patient values shown. Statistical significance was assessed using the Kruskal–Wallis test followed by Dunn's multiple‐comparison test. (C) Representative CD27 versus CD45RA plots of CD4^+^ and CD8^+^ T cells from paired peripheral blood and liver tissue of a patient with AIH. Populations were operationally defined as CD27^+^CD45RA^+^ naïve‐like, CD27^+^CD45RA^−^ central‐memory‐like (TCM‐like), CD27^−^CD45RA^−^ effector‐memory‐like (TEM‐like) and CD27^−^CD45RA^+^ terminally differentiated effector‐memory‐like (TEMRA‐like) cells. *p < 0.05, **p < 0.01, ***p < 0.001, ****p < 0.0001.
**Figure S2:** CD69/CD103‐defined T‐cell phenotypes in peripheral blood. Distribution of CD69/CD103‐defined subsets among circulating CD4^+^ (A) and CD8^+^ (B) T cells from ANTL, MASLD and AIH patients. Subsets were classified as CD69 single‐positive (CD103^−^CD69^+^), CD69^+^CD103^+^ double‐positive, CD103 single‐positive (CD103^+^CD69^−^) and double‐negative (CD103^−^CD69^−^) cells. Data represent mean ± SD, with individual patient values shown. Statistical significance was assessed using the Kruskal–Wallis test followed by Dunn's multiple‐comparison test.
**Figure S3:** Representative intracellular cytokine histograms of liver‐derived CD8+ T cells in ANTL, MASLD and AIH. Representative flow‐cytometry histograms showing IL‐17A, TNF‐α, IL‐4 and IL‐13 production by liver‐derived CD8+ T cells from ANTL, MASLD and AIH patients following PMA/ionomycin stimulation.
**Figure S4:** Median fluorescence intensity of intracellular cytokines in liver‐derived CD8^+^ T cells. Median fluorescence intensity (MFI) of IFN‐γ (A), IL‐17A (B), IL‐4 (C), IL‐13 (D) and TNF‐α (E) was quantified within the corresponding cytokine‐positive liver‐derived CD8^+^ T‐cell populations from ANTL, MASLD and AIH patients following PMA/ionomycin stimulation. Data represent mean ± SD, with individual patient values shown. Statistical significance was assessed using the Kruskal–Wallis test followed by Dunn's multiple‐comparison test. *p < 0.05.
**Figure S5:** Cytokine responses of circulating and intrahepatic CD4+ T cells in ANTL, MASLD and AIH. (A) Heatmap showing log_2_‐transformed ratios of cohort‐mean cytokine‐positive CD4+ T‐cell frequencies in liver versus peripheral blood across the ANTL, MASLD and AIH cohorts; positive values indicate higher mean frequencies in liver tissue, whereas negative values indicate higher mean frequencies in peripheral blood. (B–F) Frequencies of IFN‐γ (B), IL‐17A (C), IL‐13 (D), IL‐4 (E) and TNF‐α (F) CD4+ T cells in peripheral blood and liver tissue. Cytokine‐positive cells are expressed as percentages of CD4+ T cells. Data represent mean ± SD, with individual patient values shown. Statistical significance was assessed using the Kruskal–Wallis test followed by Dunn's multiple‐comparison test. *p < 0.05, ***p < 0.001, ****p < 0.0001.
**Figure S6:** Association of treatment status with liver enzyme levels in AIH patients. (A) Correlation between treatment status and alanine aminotransferase (ALT) levels. (B) Correlation between treatment status and aspartate aminotransferase (AST) levels. Treatment status was coded as untreated or treated and is displayed as a categorical variable. Each dot represents one patient. Spearman correlation coefficients (*ρ*) and corresponding *p* values are shown.
**Figure S7:** Associations of intrahepatic TRM‐associated and IFN‐γ‐producing CD8+ T‐cell phenotypes with inflammatory disease activity in AIH. (A and B) Correlations of intrahepatic TRM‐phenotype CD3+ T‐cell frequency with serum ALT (A) and mHAI inflammatory grade (B). (C and D) Correlations of IFN‐γ‐producing intrahepatic CD8+ T‐cell frequency with serum ALT (C) and mHAI inflammatory grade (D). (E and F) Frequencies of TRM‐phenotype CD3+ T cells (E) and IFN‐γ‐producing intrahepatic CD8+ T cells (F) in biochemically active (ALT > laboratory upper limit of normal; *n* = 15) and biochemically inactive (ALT within the normal range; *n* = 4) patients. Each dot represents one patient. Correlations were assessed using two‐sided Spearman's rank correlation; Spearman's *ρ* and corresponding *p* values are shown. Group comparisons were performed using two‐sided Mann–Whitney *U* tests. mHAI analyses included 18 patients because histological activity could not be assessed in one patient.

**Table S1:** Reagents used for flow cytometry and intracellular cytokine staining.

## Data Availability

The data that support the findings of this study are available from the corresponding author upon reasonable request.
