# Supplementary material for: Intrahepatic Tissue‐Resident CD8 + T‐Cell Programmes Associate With Fibrosis and Local Immune Remodelling in Autoimmune Hepatitis
Source: Liver Int. 2026 Oct 5;46(11):e70897. doi: 10.1111/liv.70897 (PMC13639376; doi:10.1111/liv.70897)
Supplement: Supplementary file 1 [file LIV-46-0-s002.pdf]

Figure S1

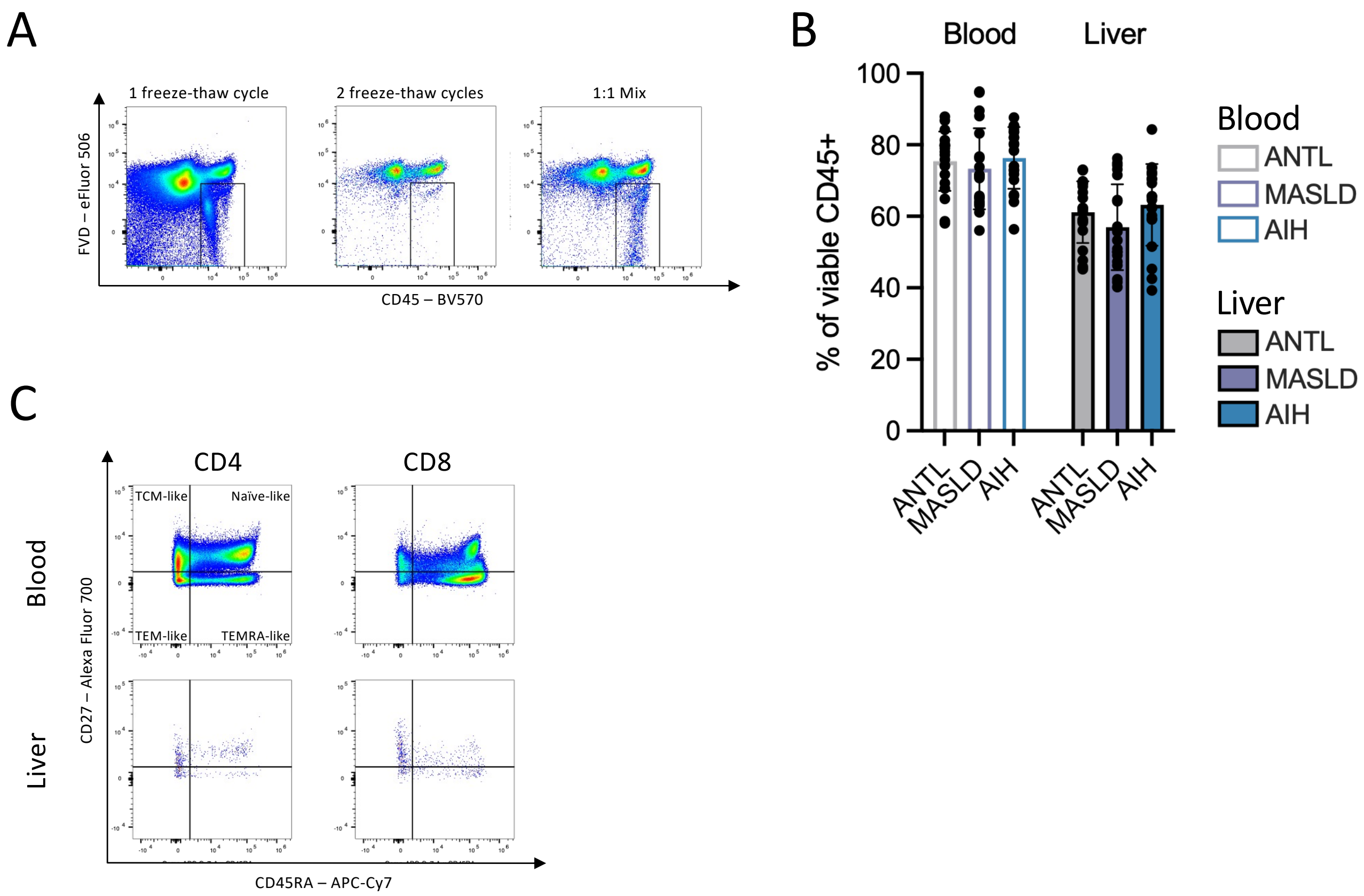
A
1 freeze-thaw cycle
2 freeze-thaw cycles
1:1 Mix
FVD – eFluor 506
CD45 – BV570
B
Blood
Liver
% of viable CD45+
100
80
60
40
20
0
ANTL
MASLD
AIH
ANTL
MASLD
AIH
Blood
ANTL
MASLD
AIH
Liver
ANTL
MASLD
AIH
C
CD4
CD8
Blood
Liver
TCM-like
Naïve-like
TEM-like
TEMRA-like
CD27 – Alexa Fluor 700
CD45RA – APC-Cy7

Figure S2

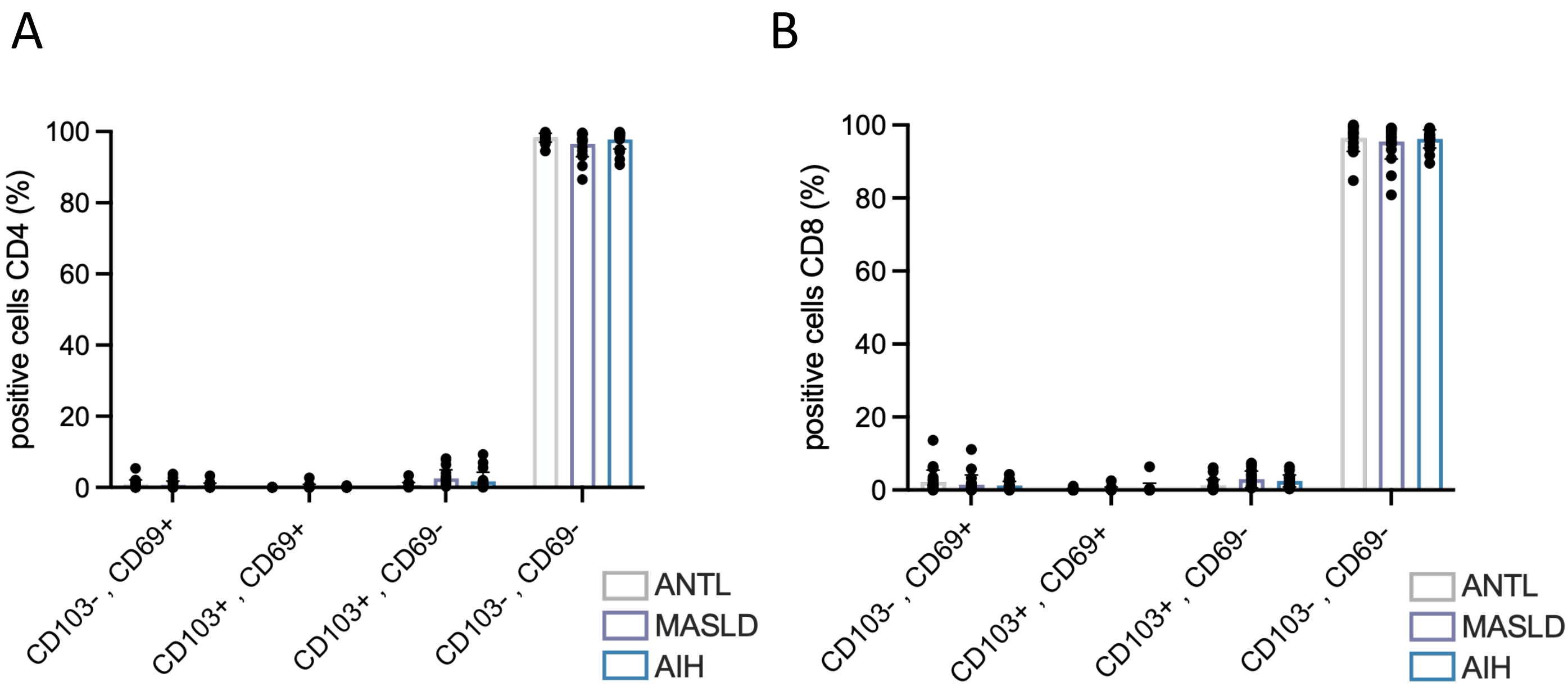

A
positive cells CD4 (%)
100
80
60
40
20
0
CD103- , CD69+
CD103+ , CD69+
CD103+ , CD69-
CD103- , CD69-
ANTL
MASLD
AIH
B
positive cells CD8 (%)
100
80
60
40
20
0
CD103- , CD69+
CD103+ , CD69+
CD103+ , CD69-
CD103- , CD69-
ANTL
MASLD
AIH

Figure S3

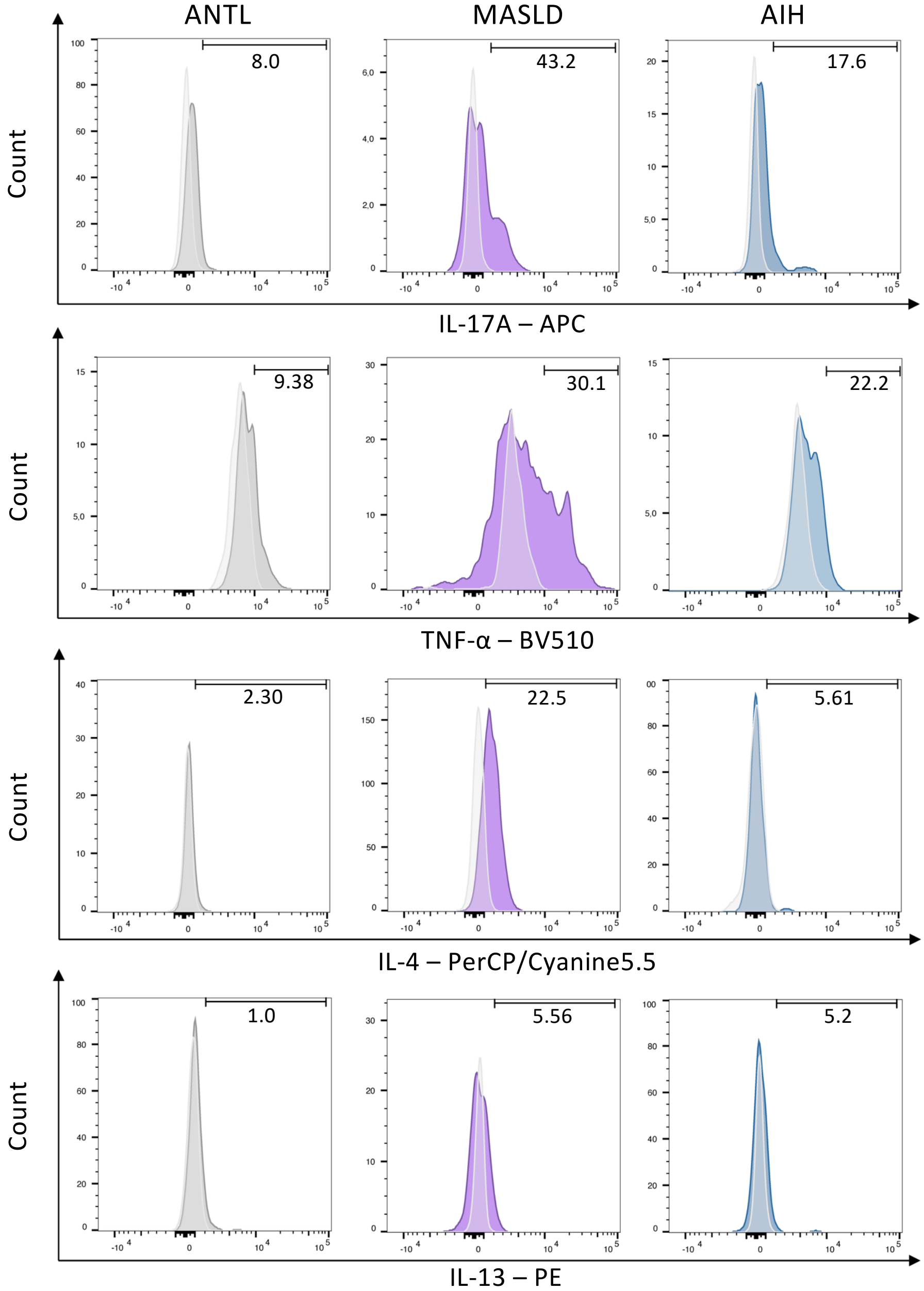

ANTL
MASLD
AIH
Count
8.0
43.2
17.6
IL-17A – APC
9.38
30.1
22.2
TNF-α – BV510
2.30
22.5
5.61
IL-4 – PerCP/Cyanine5.5
1.0
5.56
5.2
IL-13 – PE

Figure S4

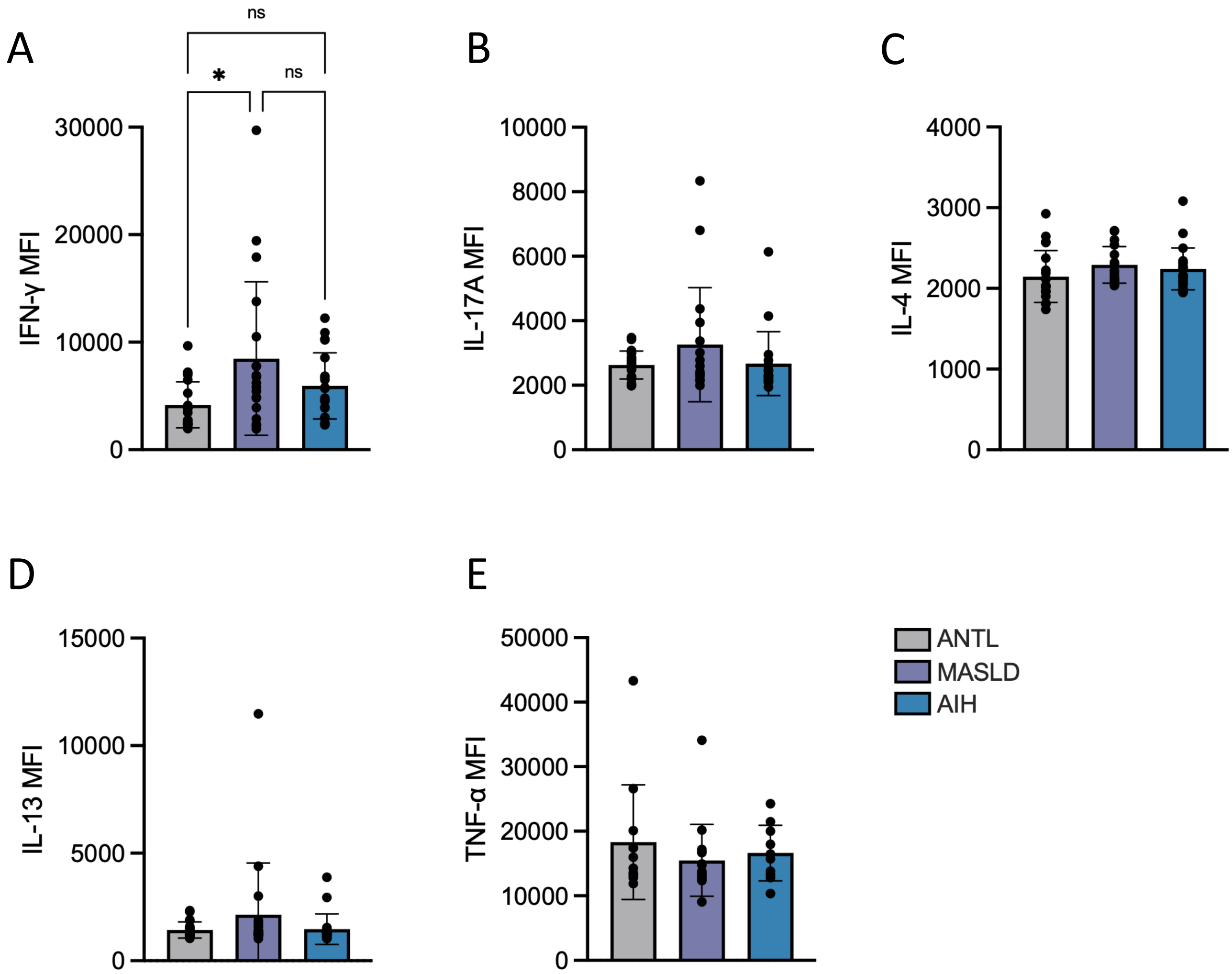

A
ns
*
ns
30000
20000
10000
0
IFN-γ MFI
B
10000
8000
6000
4000
2000
0
IL-17A MFI
C
4000
3000
2000
1000
0
IL-4 MFI
D
15000
10000
5000
0
IL-13 MFI
E
50000
40000
30000
20000
10000
0
TNF-α MFI
ANTL
MASLD
AIH

Figure S5

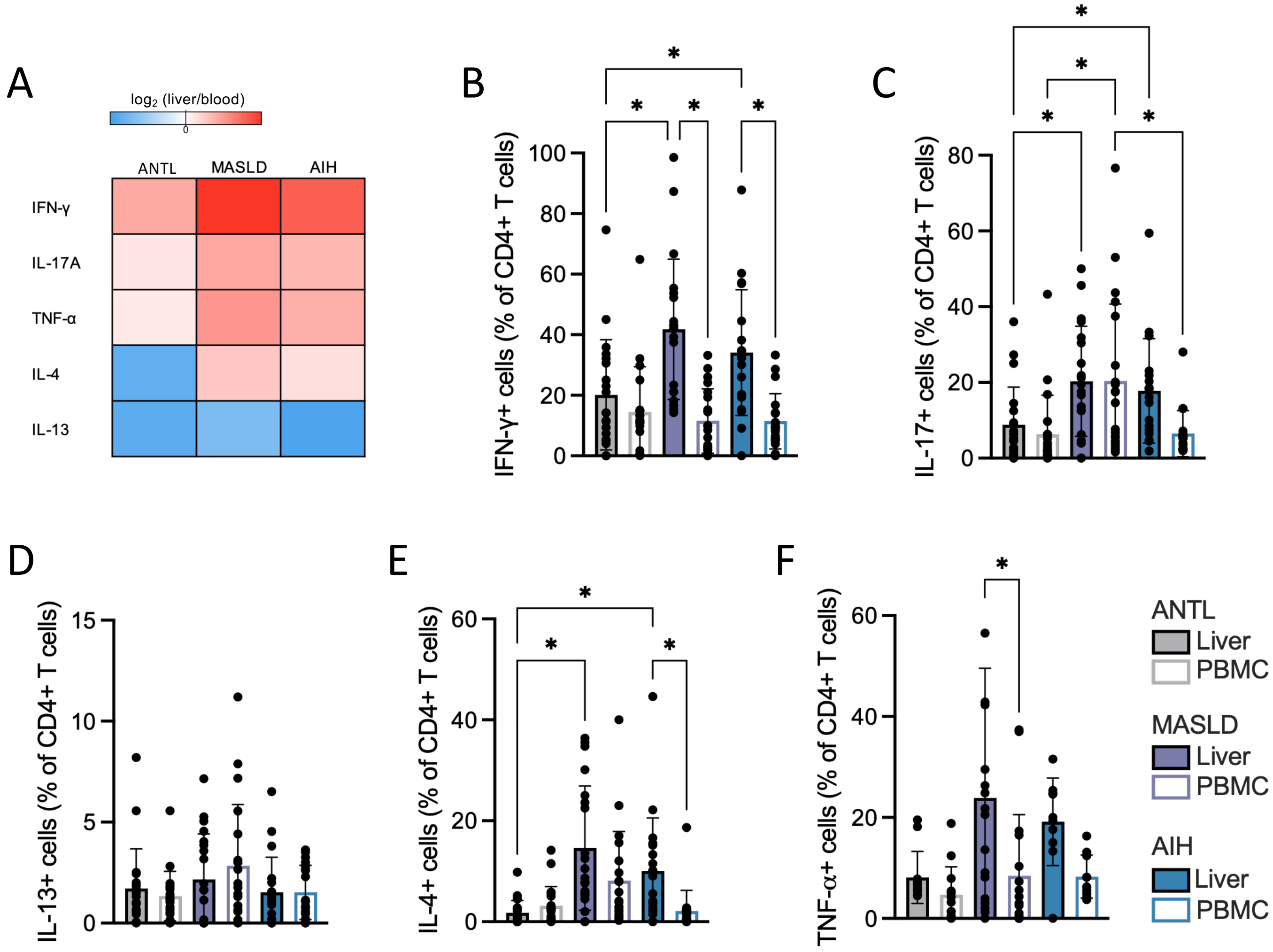
A
log2 (liver/blood)
0
ANTL
MASLD
AIH
IFN-γ
IL-17A
TNF-α
IL-4
IL-13
B
IFN-γ+ cells (% of CD4+ T cells)
C
IL-17+ cells (% of CD4+ T cells)
D
IL-13+ cells (% of CD4+ T cells)
E
IL-4+ cells (% of CD4+ T cells)
F
TNF-α+ cells (% of CD4+ T cells)
ANTL
Liver
PBMC
MASLD
Liver
PBMC
AIH
Liver
PBMC

Figure S6

A

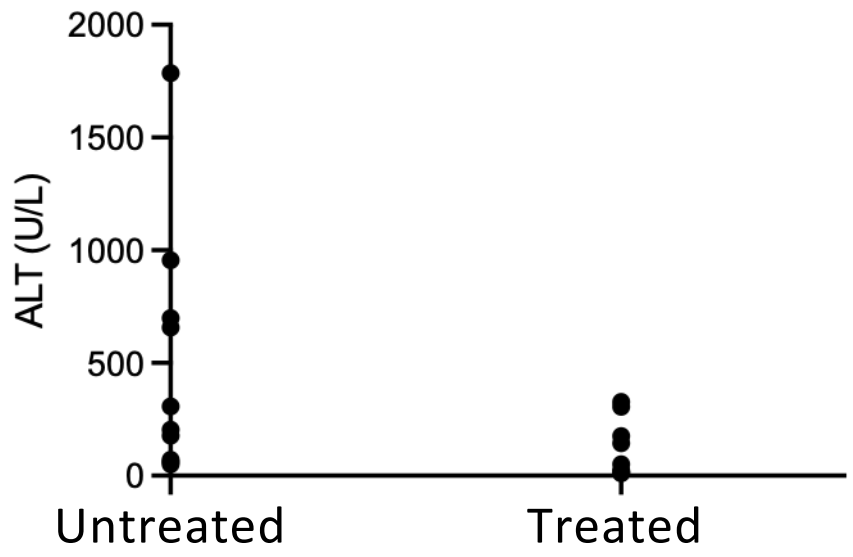

p = 0.0173
ρ = -0.5389

B

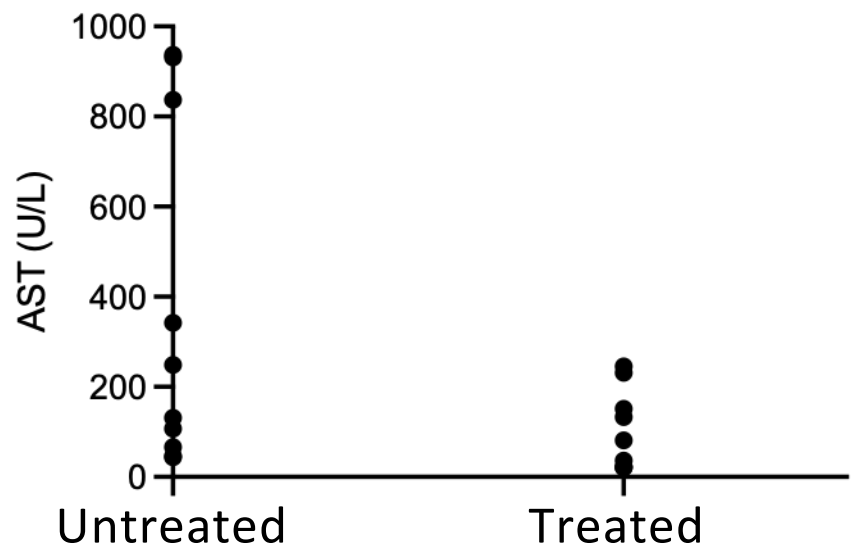

p = 0.0707
ρ = -0.4236

Figure S7

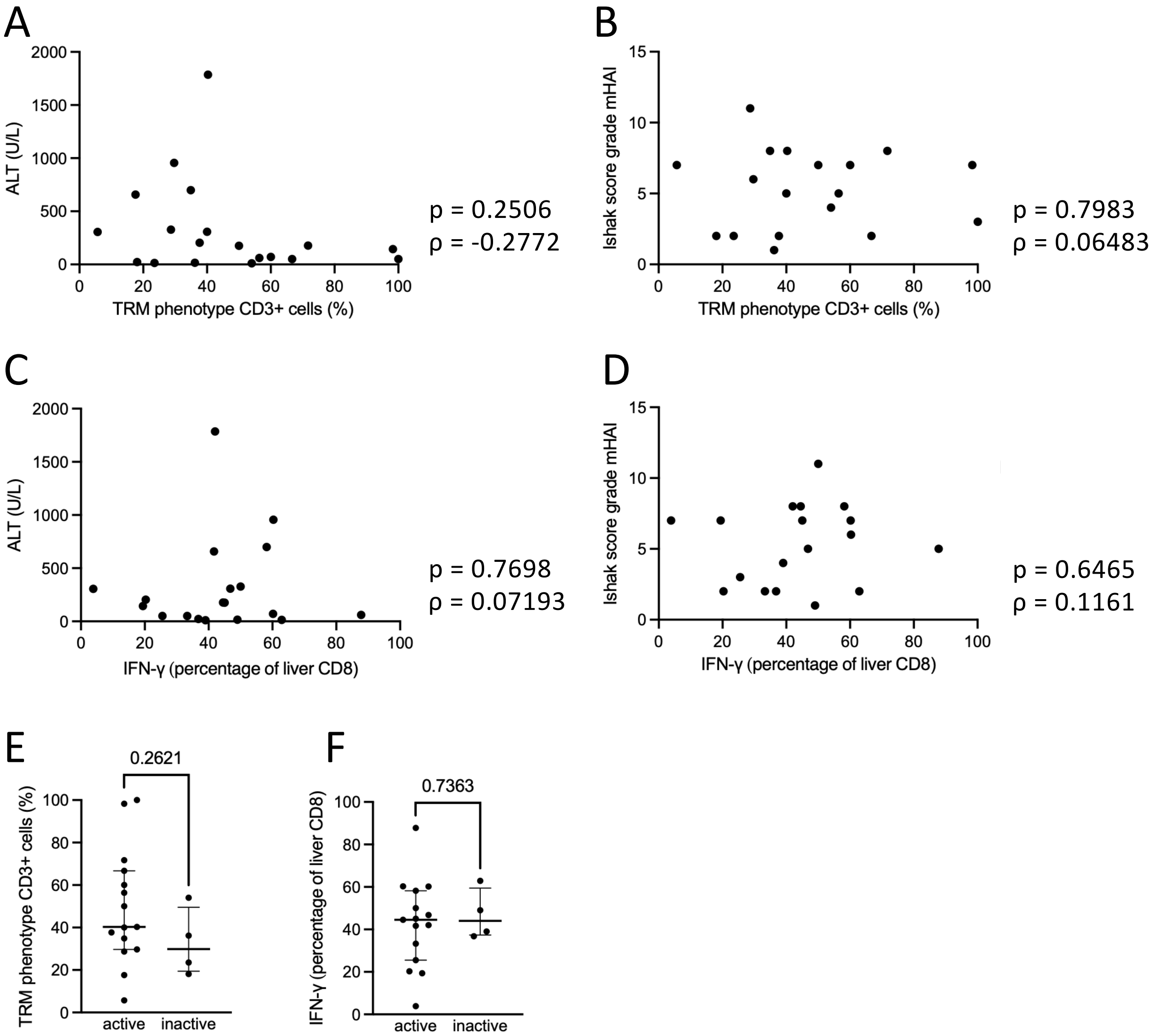

A
ALT (U/L)
TRM phenotype CD3+ cells (%)
p = 0.2506
ρ = -0.2772
B
Ishak score grade mHAI
TRM phenotype CD3+ cells (%)
p = 0.7983
ρ = 0.06483
C
ALT (U/L)
IFN-γ (percentage of liver CD8)
p = 0.7698
ρ = 0.07193
D
Ishak score grade mHAI
IFN-γ (percentage of liver CD8)
p = 0.6465
ρ = 0.1161
E
0.2621
TRM phenotype CD3+ cells (%)
active
inactive
F
0.7363
IFN-γ (percentage of liver CD8)
active
inactive
