# Supplementary material for: Intrahepatic Tissue‐Resident CD8 + T‐Cell Programmes Associate With Fibrosis and Local Immune Remodelling in Autoimmune Hepatitis
Source: Liver Int. 2026 Oct 5;46(11):e70897. doi: 10.1111/liv.70897 (PMC13639376; doi:10.1111/liv.70897)
Supplement: Supplementary file 2 [file LIV-46-0-s001.docx]

Table S1. Reagents used for flow cytometry and intracellular cytokine staining.

| **Antigen** | **Fluorochrome** | **Manufacturer** | **Catalog no.** | **Isotype** | **Clone** | **Dilution** |
| --- | --- | --- | --- | --- | --- | --- |
| FVD | eFluor 506 | Invitrogen | 65-0866-14 | - | - | 1:2000 |
| CD45 | BV570 | BioLegend | 304033 | Mouse IgG1, κ | HI30 | 1:100 |
| CD3 | PerCP | BD Biosciences | 345766 | Mouse IgG1, κ | SK7 | 1:20 |
| TCRαβ | FITC | BD Biosciences | 333140 | Mouse IgG1, κ | WT31 | 1:100 |
| TCRγδ | PerCP-eFluor 710 | Invitrogen | 46-9959-42 | Mouse IgG1, κ | B1.1 | 1:100 |
| CD4 | BV605 | BioLegend | 300555 | Mouse IgG1, κ | RPA-T4 | 1:200 |
| CD8α | BV421 | BioLegend | 301036 | Mouse IgG1, κ | RPA-T8 | 1:50 |
| CD69 | BUV395 | BD Biosciences | 564364 | Mouse IgG1, κ | FN50 | 1:100 |
| CD103 | PE/Dazzle 594 | BioLegend | 350223 | Mouse IgG1, κ | Ber-ACT8 | 1:100 |
| CXCR6 | PE/Cyanine7 | BioLegend | 356011 | Mouse IgG2a, κ | K041E5 | 1:50 |
| CD49a | Alexa Fluor 647 | BioLegend | 328310 | Mouse IgG1, κ | TS2/7 | 1:100 |
| CD27 | Alexa Fluor 700 | BioLegend | 356415 | Mouse IgG1, κ | M-T271 | 1:200 |
| CD45RA | APC/Cyanine7 | BioLegend | 304128 | Mouse IgG2b, κ | HI100 | 1:100 |
| IFN-γ | BV785 | BioLegend | 502542 | Mouse IgG1, κ | 4SB3 | 1:50 |
| TNF-α | BV510 | BioLegend | 502949 | Mouse IgG1, κ | MAb11 | 1:100 |
| IL-4 | PerCP/Cyanine5.5 | BioLegend | 500822 | Rat IgG1, κ | MP4-25D2 | 1:50 |
| IL-13 | PE | BioLegend | 501903 | Rat IgG1, κ | JES10-5A2 | 1:100 |
| IL-17A | APC | Thermo Fisher Scientific (eBioscience) | 17-7179-41 | Mouse IgG1, κ | eBio64DEC17 | 1:100 |
